# Analysis of Paediatric Clinical Trial Characteristics and Activity Over 23 Years—Impact of the European Paediatric Regulation on a Single French Clinical Research Center

**DOI:** 10.3389/fped.2022.842480

**Published:** 2022-04-26

**Authors:** Johanna Arnadottir, François Luc, Florentia Kaguelidou, Evelyne Jacqz-Aigrain, the Collaborative CIC1426 Investigator Group, C. Auvin

**Affiliations:** ^1^Clinical Investigation Center CIC1426, Hôpital Robert Debré Assistance Publique-Hôpitaux de Paris INSERM, Paris, France; ^2^Paris University, Paris, France; ^3^Department of Paediatric Pharmacology and Pharmacogenetics, Hôpital Robert Debré, Assistance Publique-Hôpitaux de Paris, Paris, France

**Keywords:** paediatric trial, drug evaluation, industrial trials, public trials, European paediatric regulation

## Abstract

As unlicensed or off-label drugs are frequently prescribed in children, the European Pediatric Regulation came into force in 2007 to improve the safe use of medicinal products in the pediatric population. This present report analyzes the pediatric research trials over 23 years in a clinical research center dedicated to children and the impact of regulation. The database of trial characteristics from 1998 to 2020 was analyzed. We also searched for differences between two periods (1998–2006 and 2007–2020) and between institutional and industrial sponsors during the whole period (1998–2020). A total of 379 pediatric trials were initiated at our center, corresponding to inclusion of 7955 subjects and 19448 on-site patient visits. The trials were predominantly drug evaluation trials (*n* = 278, 73%), sponsored by industries (*n* = 216, 57%) or government/non-profit institutions (*n* = 163, 43%). All age groups and most subspecialties were concerned. We noted an important and regular increase in the number of trials conducted over the years, with an increased number of multinational, industrially sponsored trials. Based on the data presented, areas of improvement are discussed: (1) following ethical and regulatory approval depending on the sponsor, the mean time needed for administrative and financial agreement, validation of trial procedures allowing trial initiation at the level of the center was 6.3 and 6.5 months (periods 1 and 2, respectively) and should be reduced, (2) availability of expert research teams remain insufficient, time dedicated to research attributed to physicians should be organized and recognition of research nurses is required. The positive impact of the European Pediatric Regulation highlights the need to increase the availability of trained research teams, organized within identified multicenter international pediatric research networks.

## Introduction

The licensing process was introduced to ensure that medicines are safe, effective and of high quality. Thousands of clinical trials are conducted internationally every year to evaluate new drugs, optimize dosage schedules, validate new indications, and determine efficacy and safety parameters to improve health interventions.

Many studies have shown a high proportion (up to 80%) of unlicensed or off-label use of medicines for the treatment of children and neonates, both in- and outside hospitals (1–7).

Given the frequent lack of other options, such practice remains common and guidelines for off-label prescription were published in 2014 by the Committee on Drugs of the American Academy of Pediatrics (8). More recently, in 2020, the European Academy of Pediatrics and the European Society for Developmental Perinatal and Pediatric Pharmacology issued a joint policy statement providing guidance for off-label prescriptions by health care professionals (9). Both these guidelines emphasize the importance of continued research to improve understanding of the effects of medicines in the pediatric population.

Starting in the 1950s, health care professionals and regulators identified the obvious implications of the lack of specific drug evaluation in all pediatric age groups. Several decades passed before regulations were issued for improvement, proposing a financial incentive to pharmaceutical companies. The FDA Modernization Act was the first such act and came into force in 1997 in the United States (10). This was followed by The Best Pharmaceuticals for Children Act in 2002 (11), the Pediatrics Research Equity Acts in 2003 (12) and the Newborn Drug Initiative in 2006 (13). In Europe, the European Pediatric Regulation came into force in January 26, 2007 (14). Under this new regulation, a Pediatric Investigation Plan (PIP) became mandatory for all new medicines to be authorized by the European Medicines Agency (EMA) in the European Union (EU). The objective was to improve the safe use of medicinal products in the pediatric population which has traditionally been particularly subject to unauthorized and off-label treatments. A second objective was to avoid the unnecessary exposure of children to clinical trials while ensuring that these trials are of high quality and conducted in an ethical manner.

In France, Clinical Investigation Centers (CIC) were created to answer to institutional and industrial solicitations to conduct clinical research trials (15). Our pediatric clinical investigation center at Robert Debré University Hospital (CIC-RDB) was the first CIC in France. It opened in 1992, 15 years prior to the Pediatric Regulation and interactions between researchers, pediatricians and pharmaceutical industries were progressively created to design and conduct pediatric clinical research projects.

In the present report, our aim was to review the main characteristics of the clinical trials conducted in our research center over a 23-year period from 1998 to 2020 and analyze the changes that occurred between two periods (1998–2006 and 2007–2020) before and after the introduction of the Pediatric Regulation in 2007. We also searched for differences between institutional and industrial sponsors.

## Materials and Methods

### Data Sources

We accessed the data included in our in-house database of clinical trials conducted at the CIC-RDB. This database is maintained in a prospective manner by FL and includes all research projects conducted at our center since its opening. Data were verified by JA and whenever possible, missing data were retrieved in the original paper documents.

The database contains information on the medical specialty and objectives of the trial, the type of trial and phase, subject age groups, the intended and actual number of participants included in the trial at our center, type of sponsor, whether the trial is a single- or multicenter trial, conducted on the national, European, or international level. In addition, information is collected on key dates such as the time required for trial initiation (i.e., trial open to recruitment) after completion of regulatory requirements (if necessary), local administrative and financial approval at the level of the center and procedures for trial conduct, as well as dates of first and last study subject visits.

### Data Analysis

We performed a first analysis of all trials opened and conducted between January 1, 1998 and December 31, 2020. We did not include the first 6 years of activity at CIC-RDB (1992–1997), corresponding to the first years of organization and training.

In addition, data were compared between two time-periods: period 1 from January 1, 1998 to December 31, 2006 and period 2 from January 1, 2007 to December 31, 2020. The Pediatric Regulation came into force on January 26, 2007. No trials were opened at our site from January 1 to 25, 2007 thus the two periods are separated in time by the application of the Pediatric Regulation.

Thirdly, we compared trial characteristics with respect to the type of sponsor of the trial. For this analysis we looked at all trials in our database from January 1, 1998 and December 31, 2020 and separated trials sponsored by the pharmaceutical industry and institutional sponsors. The second category included all governmental and not-for-profit organizations as well as all other non-industrial sponsors.

Lastly, we analyzed the number of subject visits, the number of trial inclusions and the number of open trials by year. For this analysis, we assessed the period from January, 1 1998 at which date there were 22 trials recruiting, to December 31, 2020.

## Results

### Overall Activity

#### Trial Characteristics

During the 23-years from January 1, 1998 to December 31, 2020, a total of 379 clinical trials were initiated at CIC-RDB, corresponding to the inclusion of 7955 subjects and 19448 on-site patient visits (Table 1).

**TABLE 1:** Trial characteristics at the time of trial opening at CIC-RDB for trials initiated from January 1, 1998 to December 31, 2020.

	01/01/1998 - 31/12/2020		“Period 1” 01/01/1998–31/12/2006		“Period 1” Number per year	“Period 2” 01/01/2007–31/12/2020		“Period 2” Number per year
Trials opened (*n*)	379		113		12.6	266		19
Type of sponsor (n*)	379		113			266		
Industry	216	57%	44	39%	4.9	172	65%	12.3
Government*/not-for-profit organizations	163	43%	69	61%	7.7	94	35%	6.7
Aim (n*)	379		113			266		
Drug evaluation	278	73%	60	53%	6.7	218	82%	15.6
Efficacy-Safety	258	93%	50	83%	5.6	208	95%	14.8
Pharmacokinetics	147	53%	24	40%	2.7	123	56%	8.8
Both	126	45%	16	27%	1.8	110	50%	7.9
Physiology-Pathology	101	27%	53	47%	5.9	48	18%	3.4
Planned age groups (n*)	333*^a^*		77			256		
[0–29 d[	50	15%	6	8%	0.7	44	17%	3.1
[29 d–2 y[	157	47%	35	45%	3.9	122	48%	8.7
[2 y–6 y[	194	58%	43	56%	4.8	151	59%	10.8
[6 y–12 y[	260	78%	59	77%	6.6	201	79%	14.4
[12 y–18 y[	262	79%	58	75%	6.4	204	80%	14.6
[18 y]	153	46%	41	53%	4.6	112	44%	8.0
Location (n*)	379		113			266		
Single Center	47	12%	36	32%	4.0	11	4%	0.8
Multi Center	332	88%	77	68%	8.6	255	96%	18.2
National	101	30%	37	48%	4.1	64	25%	4.6
European	31	9%	1	1%	0.1	30	12%	2.1
International	200	60%	39	51%	4.3	161	63%	11.5
Design (n*)	379		113			266		
Cohort	25	7%	3	3%	0.3	22	8%	1.6
Clinical trial	346	91%	108	95%	12.1	238	90%	17.0
Comparative	160	42%	38	34%	4.2	122	46%	8.7
Randomized	151	40%	31	27%	3.4	120	45%	8.6
Blinded/double-blinded	110	29%	22	19%	2.4	88	33%	6.2
Medical device	8	2%	2	2%	0.2	6	2%	0.4
Medical specialties (*n*)	379		113			266		
Neurology-psychiatry	66	17%	14	12%	1.6	52	20%	3.7
Endocrinology	53	14%	29	26%	3.2	24	9%	1.7
Hematology-hemato-oncology	65	17%	11	10%	1.2	54	20%	3.9
Nephrology	53	14%	11	10%	1.2	42	16%	3.0
Immunology-infectious diseases	39	10%	11	10%	1.2	28	11%	
Pneumology	42	11%	13	12%	1.4	29	11%	2.1
Gastro-enterology-nutrition	28	7%	13	12%	1.4	15	6%	1.1
Obstetrics–neonatology	8	2%	1	1%	0.1	7	3%	0.5
Anesthesia-surgery	6	2%	2	2%	0.2	4	2%	0.3
Syndromic diseases	6	2%	3	3%	0.3	3	1%	0.2
Other (dermatology, ENT, reference values)	13	3%	5	4%	0.6	8	3%	0.6
Planned length of study per participant in years (*n*)	355*^a^*		101			254		
[0–1[	214	60%	72	71%	8.0	142	56%	10.1
[1–2[	61	17%	17	17%	1.9	44	17%	3.1
[2–4[	52	15%	6	6%	0.7	46	18%	3.3
[4	28	8%	6	6%	0.7	22	9%	1.6
Anticipated number of participants per trial (*n*)	319^ a^		109			210		
[0–11[	215	67%	45	41%	8.0	170	81%	12.1
[11–51[	70	22%	42	39%	4.7	28	13%	2.0
[51–100[	23	7%	14	13%	1.6	9	4%	0.6
≥100	11	3%	8	7%	0.9	3	1%	< 0.1
Approval timeline (*n*) in months (mean)								
Between ethics approval—initiation	326*^a^*	6.3	113*^a^*	5.7	/	213*^a^*	6.6	/
Between Regulatory approval—initiation	237*^a^*	6.5	25*^a^*	2.5	/	212*^a^*	7.0	/
Between initiation—first inclusion	298*^a^*	4.3	100*^a^*	3.6	/	198*^a^*	4.6	/

^a^Number of trials with available information. **Government: all governmental institutions. ***d, days; y, years.

The aim of 278 trials (73%) was drug evaluation with a focus on efficacy and safety (*n* = 258) or pharmacokinetics (*n* = 147) as well as trials with both these objectives (*n* = 126), while 101 trials (27%) studied physiology and/or the physio-pathology of pediatric diseases.

These trials were sponsored by the pharmaceutical industry (*n* = 216) or governmental and not-for-profit organizations (*n* = 163). Most were multicenter trials (*n* = 332, 88%) and the majority were conducted internationally (*n* = 200, 60%).

All age groups were represented, with a majority of participants planned between the ages of 6 to 18 years. Some projects also planned for inclusion of adults and pediatric subjects (*n* = 153, 46%) and some did not exclude inclusions during the neonatal period (*n* = 50, 15%).

Sixty percent (*n* = 214) of the trials planned for a duration of inclusion for each subject of less than 1 year whereas 8% (*n* = 28) planned for more than 4 years of study follow-up. Sixty-seven percent (*n* = 215) of the trials planned to recruit 10 or fewer participants at our site and 22% (*n* = 70) between 11 and 50 subjects.

Additional characteristics of the research projects are presented in Table 1.

#### Key Timelines

The mean time between ethical approval and trial initiation at our site was 6.3 months (*n* = 326), while 6.5 months passed between the regulatory approval to trial initiation (*n* = 237). The first subject was included a mean 4.3 months after trial initiation (*n* = 298).

A total of 86% (*n* = 202) of the trials took more than 1 year for completion at our site with 40% (*n* = 94) lasting between 2 and 4 years and 21% (*n* = 48) having a study duration of more than 4 years.

Of the 379 clinical trials opened during the 23-year study period, 234 (62%) were closed by the sponsor (Table 2). A mean time of 9.6 months passed between the last visit of the last participant included and trial closure by the sponsor. For 12 additional trials, the last visit of the last patient included had been completed at our site and these trials were awaiting closure by the sponsor at the end of the period of analysis.

**TABLE 2:** Analysis of trials closed at CIC-RDB during the study period (January 1, 1998–December 31, 2020) and during period 1 (1998–2006) and period 2 (2007–2020).

	Trials opened during 01/01/1998–31/12/2020 (*n* = 379)		Trials opened during “period 1” 01/01/1998–31/12/2006 (*n* = 113)		Trials opened during “period 2” 01/01/2007–31/12/2020 (*n* = 265)	
Trials closed by the sponsor (*n*)	234		103		131	
Trials finalized at CIC-RDB* (*n*)	246		101		145	
Last visit of last participant to trial closure [n of trials, months (mean)]	225	9.6	93	5.7	131	11.9
Length of trials at CIC-RDB in years (*n*)	234		103		131	
[0–1[	32	14%	14	14%	18	14%
[1–2[	60	26%	23	22%	37	28%
[2–4[	94	40%	44	43%	50	38%
≥4	48	21%	22	21%	26	20%
Percentage of inclusions at CIC-RDB** (*n*)	207*^a^*		99		108	
0%	34	16%	8	8%	26	24%
]0%–50%[	44	21%	24	24%	20	19%
[50%–100%[	78	38%	43	43%	35	32%
≥100%	51	25%	24	24%	27	25%
Number of patients included (*n*)	7955		3591		4364	
Number of visits at our center (*n*)	19454		4893		14561	

^a^Number of trials with available information *Trials are finalized in our center when the last visit of the last included patient has been completed. **The percentage of inclusions at the Clinical investigation center of Robert Debré Hospital (CIC-RDB) is calculated from the number of included patients anticipated at the trial opening.

Among the trials closed by the sponsor, 38% (*n* = 78) included at least half of the intended number of participants and 25% (*n* = 51) surpassed the initial objective, while 16% (*n* = 34) were closed by the sponsor without including a single subject at our center.

### Comparison of Trial Characteristics Between Period 1 (1998–2006) and Period 2 (2007–2020)

A total of 113 and 266 trials opened during periods 1 and 2, respectively. This represents a 51% increase between the two periods with a mean of 12.6 and 19.0 trial initiations per year during periods 1 and 2, respectively.

The proportion of trials sponsored by the pharmaceutical industry increased: they were 39% of all trials during period 1 (*n* = 44) and 65% of all trials in period 2 (*n* = 172) and assumed to be validated by a Pediatric Investigation Plan (although this item was not recorded in the database), 53% (*n* = 60) during period 1 focused on drug evaluation compared to 82% (*n* = 218) during period 2. The proportion of physiology or physio-pathology trials decreased accordingly from 47% during period 1 (*n* = 53) to 18% in period 2 (*n* = 48).

**FIGURE 1:**
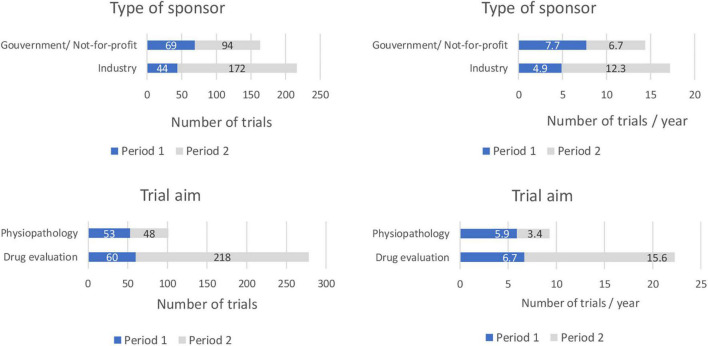
Comparison of selected trial characteristics between period 1 (1998–2006) and period 2 (2007–2020). (Number of trials/and number of trials per year according to the type of sponsor, trial aim, location and design).

The proportion of multicenter trials opened during period 1 was 68% (*n* = 77). This proportion increased to 96% (*n* = 255) during period 2. Of these multicenter trials, 51% (*n* = 39) were international trials during period 1 compared to 63% (*n* = 161) during period 2 (Tables 1, 2 and Figure 1).

#### Key Timelines

The mean time passed between the ethical approval and initiation of the clinical trial at our center increased from 5.7 months in period 1 (*n* = 113) to 6.6 months in period 2 (*n* = 213), corresponding to a 16% increase. The mean time passed between the regulatory approval and initiation of the clinical trial at our center was 2.5 months for period 1 (*n* = 25) compared to 7.0 months for period 2 (*n* = 212) corresponding to a 178% increase. The mean time between trial initiation and the first inclusion at our site was 3.6 and 4.6 months, respectively for period 1 (*n* = 100) and period 2 (*n* = 198), corresponding to 31% increase.

A total of 71% (*n* = 72) of trials planned for subject trial-participation of less than 1 year among the trials opened during period 1 compared to 56% (*n* = 142) of those opened during period 2

Overall, 41% (*n* = 45) of the studies initiated during period 1 intended to recruit 10 or fewer subjects and 39% between 11 and 50 subjects (*n* = 42). During period 2, 81% of the trials initiated planned for 10 or fewer inclusions (*n* = 170) and 13% between 11 and 50 subjects (*n* = 28).

As presented in Table 2, **91**% of the trials opened during period 1 were closed by the sponsor (*n* = 103). 49% of those opened during period 2 were closed (*n* = 145), an additional 14 trials had been finalized and were awaiting trial closure at the end of the period of analysis and 120 trials were still ongoing.

Among the trials opened during period 1, 43% had recruited at least half of the intended number of subjects at the time of closure (*n* = 43), 24% had surpassed this objective (*n* = 24) and 8% closed without including a single participant at our site (*n* = 8). At the time of closure, among the trials that were opened during period 2, 32% had recruited at least half of the intended number of participants, 25% had surpassed their initial goal and 24% closed without an inclusion (*n* = 26).

A total of 4,893 and 14,555 trial visits were conducted during period 1 and period 2, respectively. This represents a mean of 544 visits per year during period 1 and 1040 visits per year during period 2, a 91% increase in the number of cumulative annual visits between the two periods.

Additional trial characteristics at trial initiation and closing between the two periods studied can be found in Table 2.

### Comparison of the Trial Characteristics According to the Type of Sponsor

A total of 57% (*n* = 216) of all trials conducted at our center were sponsored by the pharmaceutical industry and 43% (*n* = 163) had a sponsor from governmental institutions or not-for-profit organizations. Ninety-six percent of the industrially sponsored trials focused on drug evaluation (*n* = 208) as did 43% of the institutional trials (*n* = 70). Four percent (*n* = 8) of industrially sponsored trials and 57% (*n* = 93) of institutional trials studied physiology and physio-pathology.

**FIGURE 2:**
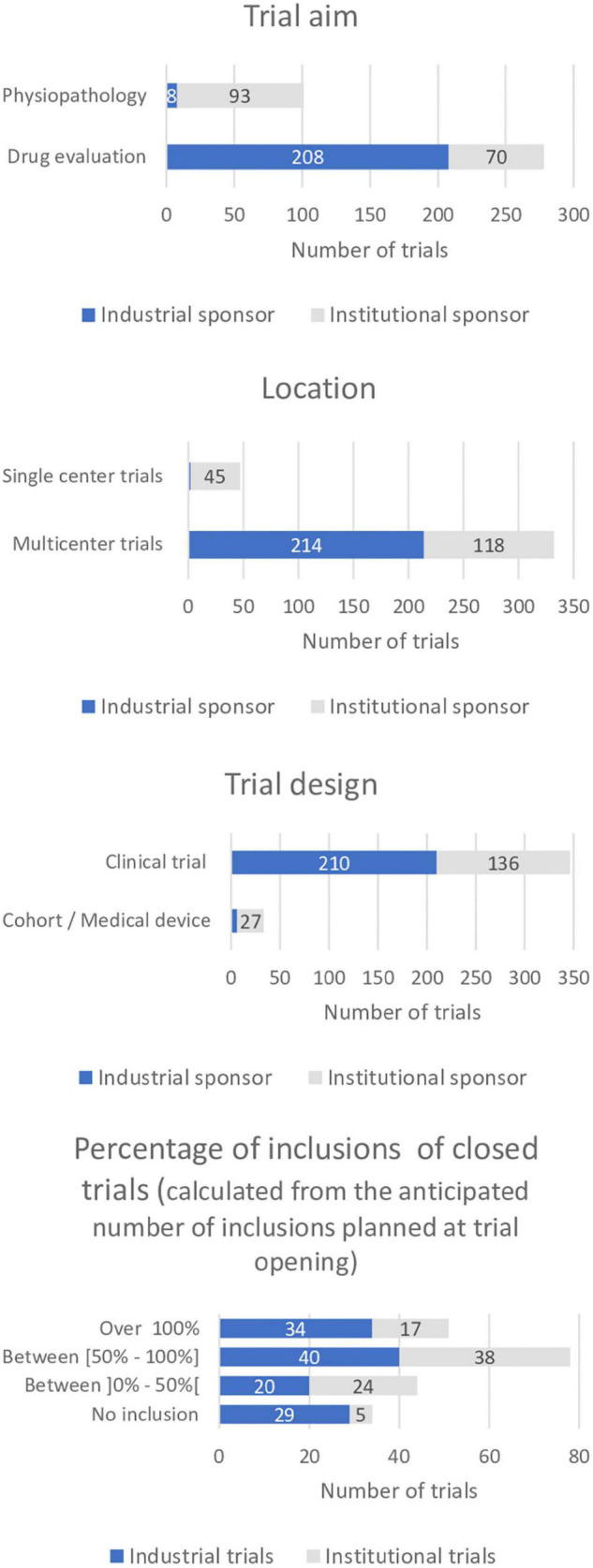
Comparison of selected trial characteristics between industrial and institutional trials. (Trial aim, location, design, percentage of inclusions in closed trials, i.e., trials closed to inclusion in the center).

Ninety-nine percent (*n* = 214) of the industrial trials were multicenter trials and 89% (*n* = 190) were conducted internationally. In comparison, 28% of institutional trials were single center (*n* = 45) and 72% multi center (*n* = 118). Eight percent (*n* = 10) of institutional multicenter trials were international trials (*n* = 10) (Table 3 and Figure 2).

**TABLE 3:** Comparison of the characteristics of trials undertaken from January 1, 1998 to December 31, 2020 at CIC-RDB sponsored by the pharmaceutical industry or institutions (governmental or not-for-profit organizations).

	All sponsors		Industrial sponsor		Institutional sponsor	
Trials opened (*n*)	379		216	57%	163	43%
Trial aim (*n*)	379		216		163	
Drug evaluation	278	73%	208	96%	70	43%
Efficacy-safety	258	93%	191	92%	67	96%
Pharmacokinetics	147	53%	116	56%	31	44%
Both	126	45%	104	50%	22	31%
Physiology-physiopathology	101	27%	8	4%	93	57%
Location (*n*)	379		216		163	
Single Center	47	12%	2	1%	45	28%
Multi Center	332	88%	214	99%	118	72%
National	101	30%	10	5%	91	77%
European	31	9%	14	7%	17	14%
International	200	60%	190	89%	10	8%
Medical specialties (*n*)	379		216		163	
Neurology and psychiatry	66	17%	39	18%	27	17%
Endocrinology	53	14%	17	8%	36	22%
Hematology—hemato-oncology	65	17%	47	22%	18	11%
Nephrology	53	14%	41	19%	12	7%
Pneumology	42	11%	31	14%	11	7%
Immunology—infectious diseases	39	10%	19	9%	20	12%
Gastro-enterology—nutrition	28	7%	13	6%	15	9%
Obstetrics—neonatology	8	2%	1	< 1%	7	4%
Anesthesia—surgery-intensive care	6	2%	2	1%	4	2%
Syndromic diseases	6	2%	1	< 1%	5	3%
Other (dermatology, ENT, reference values)	13	3%	5	2%	8	5%
Design (*n*)	379		216		163	
Cohort	25	7%	3	1%	22	13%
Clinical trial			210		136	
Comparative	160	42%	104	48%	56	34%
Randomized	151	40%	107	50%	44	27%
Blinded/Double-blinded	110	29%	84	39%	26	16%
Medical device	8	2%	3	1%	5	3%
Planned age groups* (*n*)	333*^a^*		195		138	
[0–28 d[	50	15%	28	14%	22	16%
[28 d–2 y[	157	47%	102	52%	55	40%
[2 y–6 y[	194	58%	118	61%	76	55%
[6 y–12 y[	260	78%	157	81%	103	75%
[12 y–18 y[	262	79%	159	82%	103	75%
≥18 y	153	46%	84	43%	69	50%
Planned length of study per participant in years (*n*)	355*^a^*		202		153	
[0–1[	214	60%	119	59%	95	62%
[1–2[	61	17%	32	16%	29	19%
[2–4[	52	15%	32	16%	20	13%
≥ 4	28	8%	19	9%	9	6%
Anticipated number of participants per trial (*n*)	319*^a^*		195		124	
[0–11[	215	67%	173	89%	42	34%
[11–51[	70	22%	20	10%	50	40%
[51–100[	23	7%	1	1%	22	18%
≥100	11	3%	1	1%	10	8%
Approval timeline (*n*) in months (mean)						
Between ethics—initiation	326^ a^	6.3	181	4.6	145	8.4
Between regulatory agreement—initiation	237^ a^	6.5	159	5.5	78	8.6
Between initiation—first inclusion	298^ a^	4.3	157	4.0	141	4.6
Trials closed by the sponsor (*n*)	234		135		99	
Trials finalized at CIC-RDB** (*n*)	246		124		122	
Last visit of last participant to trial closure [*n*, months (mean)]	192	9.6	100	11.1	92	7.9
Length of study at CIC-RDB in years (*n*, closed trials)	234*^a^*		135		99	
[0–1[	32	14%	22	16%	10	10%
[1–2[	60	26%	43	32%	17	17%
[2–4[	94	40%	48	36%	46	46%
≥4	48	21%	22	16%	26	26%
Percentage of inclusions at CIC-RDB*** (*n*)	207*^a^*		123		84	
0%	34	16%	29	24%	5	6%
]0–50%[	44	21%	20	16%	24	29%
[50–100%[	78	38%	40	33%	38	45%
≥100%	51	25%	34	28%	17	20%

^a^Number of trials with available information. *d, days; y, years. **Trials are finalized in our center when the last visit of the last included patient has been completed. ***The percentage of inclusions at the Clinical investigation center of Robert Debré Hospital (CIC-RDB) is calculated from the number of included patients anticipated at the trial opening.

#### Key Timelines

With regards to industrially sponsored trials, the mean time between the ethical approval and trial initiation was 4.6 months (*n* = 181). A mean time of 5.5 months passed between the regulatory approval and trial initiation (*n* = 159) and a mean time of 4.0 months between trial initiation and the inclusion of the first subject at CIC-RDB (*n* = 157). As for institutional trials, the mean time between the ethics approval and trial initiation was 8.4 months (*n* = 145). A mean time of 8.6 months was needed between the regulatory approval and trial initiation (*n* = 78) and a mean 4.6 months between trial initiation and the inclusion of the first subject at CIC-RDB (*n* = 141).

Overall, 63% (*n* = 135) of industrial trials and 61% (*n* = 99) of institutional trials had been closed by the sponsor at the end of the period of analysis. The mean time between the last visit of the last participant included at our site at local trial closure was 11.1 months for industrial trials (*n* = 100) and 7.9 months for institutional trials (*n* = 92).

Also, 33% of the industrially sponsored trials included more than half of the intended number of subjects (*n* = 40) as did 45% of the institutional trials (*n* = 38). Twenty-four percent of the industrially sponsored trials (*n* = 29) and 6% of institutional trials (*n* = 5) had been closed by the sponsor without including a trial subject at CIC-RDB.

### Clinical Investigation Center at Robert Debré University Hospital Activity Presented Annually

At the beginning of the period of analysis in 1998, 22 trials were open to inclusion. This number increased to 105 open trials at the end of 2020. Between the years 1998 and 2010, the number of trials open to inclusion at our site remained relatively stable between 22 and 44 trials and a gradual increase was seen between 2010 and 2020.

The cumulated number of patients included our center per year oscillated between a minimum of 41 to a maximum of 611. Except for the years 1998, 2019 and 2020, the annual number of subject inclusions exceeded 200 each year.

The annual cumulative number of visits varied between a minimum of 41 in 1998 to a maximum of 1,308 in 2011. Since the year 2001, the annual number of subject visits at CIC-RDB has surpassed 600 per year.

For trials open to inclusion, the mean number of visits per patient included was 1.9 in 1998 and 6.54 in 2020, illustrating that trial complexity, workload per trial and patients’ involvement increased over the years.

## Discussion

The present report is based on the analysis of the research trials conducted at the pediatric Clinical Investigation Center (CIC) of Robert Debré University Hospital in Paris, France over 23 years from January 1, 1998 to December 31, 2020. We noted an overall important and regular increase in the number of trials and analyzed their main characteristics. We also searched for differences between two periods (prior to and following the entry into force of the EU Pediatric Regulation in 2007). We noted differences in the type of sponsorship, in trial characteristics and an increase in the time required to initiate the trials. Altogether these factors affect the efficacy of pediatric clinical research and, ultimately, the children participating in the trials and their families.

To facilitate trial management and conduct, CICs were created in France, under the joint direction of the University Hospitals where they are located and The National Institute for Health and Medical Research (INSERM). CICs are open to investigators and sponsors from academic, institutional, and industrial backgrounds to conduct translational and clinical research. They are organized as clinical and research departments, with hospitalization capacities, run by an expert team dedicated to translational and clinical research.

The first CIC opened in Paris in 1992 at Robert Debré University Hospital and since then, this CIC is dedicated to pediatric research. We therefore had fifteen years of experience in conducting pediatric clinical research when the EU Pediatric Regulation entered into force in 2007. The objective of the Pediatric Regulation is to promote high quality, ethical research into medicines for children and thus increase the availability of authorized medicines for children. Briefly, the regulation is grounded on obligations balanced by incentives: a Pediatric Investigation Plan (PIP) including pediatric clinical trials, agreed by the European Medicines Agency (EMA), is required for all new medicines under investigation. A waiver can be granted for several reasons: if the medicine is likely to be ineffective or unsafe for pediatric use, if no significant therapeutic benefit is expected or if the treatment is intended for diseases not affecting the pediatric population. As a financial incentive, the pharmaceutical company can receive a 6 month extension of patent protection for the medicinal product upon completion of all the requirements of the PIP.

In 2017, the Pediatric Committee of the European Medicines Agency (EMA) presented its 10-year report on the experience acquired since the application of the Pediatric regulation (16) also summarized by Tomasi et al. (17). Their report shows that between 2007 and 2016, 950 PIPs were validated by the EMA while 486 waivers were granted. A total of 273 new medicines and 43 additional pharmaceutical forms appropriate for use in children were authorized in the EU during their period of analysis. In the report, two time-periods were compared, the 3 years immediately preceding the entry into force of the Pediatric Regulation (2004–2006) and the last 3 years before the preparation of the 10-year Report (2012–2014) (16). In addition, increased information for the pediatric population is now included in the notice for a greater number of medicines. In particular, 26% of all PIPs included the neonatal period. A positive effect is reported on oncology drug development, but with “a limited number of new medicines authorized for cancer in children” (16, 18, 19). Based on the increased number of clinical trials performed as a routine part of the development of new treatments and the number of medicines available for children, the report concludes that the Pediatric Regulation has had a “very positive impact on pediatric drug development.” However, in the same year as the EMA 10-year report, a study of off-label use of medicinal products in the EU was published (7), showing that in these 10 years, off-label prescribing in pediatrics remained equally prevalent.

In the present review of activity covering 23 years, including 10 years preceding the Pediatric Regulation, our positive results in terms of activity are in agreement with the conclusions of the EMA report (18–20). We observed a steady increase in the number of trials conducted at our center. These trials predominantly aimed at drug evaluation, half of them were multicenter, international, industry trials and concerned all age groups and most pediatric subspecialties. When comparing two periods, prior to and following the entry into force of the Pediatric Regulation, we saw at our center, a steady gradual increase in the number of open trials in period 2, along with an increased number of patients included and a greater number of on-site visits. These trials were primarily industry-sponsored, with a higher proportion of international multicenter trials during period 2. During both periods, most pediatric subspecialties were concerned, as experts, clinical wards and medical consultations in these subspecialties are available at Robert Debré University Hospital and at our center.

As noted by many authors, further time is needed to fulfill the important objectives of the Pediatric Regulation. Significant initiatives have been undertaken at the European level to advance pediatric drug development including EPTRI (European Pediatric Translational Research Infrastructure) (21) and PEDCRIN (Pediatric Clinical Research Infrastructure Network) (22) and substantial financial support has been established to set up pediatric clinical research networks dedicated to trial management and to promote collaboration at the European and international level (23–25). However, difficulties still need to be overcome to harmonize methodological (26), regulatory (27), economic (28) and ethical (29) approaches to pediatric trials and validate tools for pediatric trial conduct (22, 30–32). In addition, in our opinion, two difficulties require particular attention:

1) The availability of trained and well recognized research teams at local, national and European levels remains insufficient (33). Today, our research team is composed of 3 fulltime pediatricians (two specialized in pediatric pharmacology, particularly needed to design institutional drug trials) and one fellow. Their tasks include, but are not limited to, setting-up close collaborations/contacts with experts in paediatric subspecialties, trial evaluation and organization of trial conduct surveillance of patients’ care, (2) a nursing staff of five specialized nurses including one nursery nurse and two caregivers to take care of sick children of many different subspecialties and trained to interact with families during the trial, (3) at least five research technicians, confirmed or in training, (4) 1 lab technician and quality expert (5) 1 scientific secretary, also interacting with sponsors, regulatory and ethical bodies.
While the annual number of subject inclusions has not increased overall, we note an increased number of annual trial visits, going hand in hand with the cumulated increase in the number of open trials at our site. This inevitably results in a higher workload.
At each site, trial conduct includes the time needed to obtain parental consent, visit and sample planning, case report management and organization. The time required for these steps is nearly always underestimated. In our experience, the division of financial resources is often in favor of trial set-up and management at the expense of trial conduct at the site level. In this context, one challenge in trial execution is the limited availability of well-trained medical and nurse staff dedicated to research. For example, at our CIC, although research is one important mission attributed to physicians, time dedicated to research, i.e., “without medical duties,” is not officially organized. In a European Delphi survey aiming to collect research experience from neonatologists and identify areas for improvement, dedicated doctors were only available at 50–60% of centers, with limited possibility to reduce clinical work, while a correlation can be seen between the amount of time a physician dedicates to clinical research and the number of subjects recruited for research studies (30). A second challenge is related to “recognition of pediatric research nurses”. Nurses become “research nurses” mainly by personal and professional implication in research. Specific trainings for research nurses do exist worldwide and examples, among others, include a university training for nurses in France (34), in Europe (35), or in the United States (36). To our knowledge, the only specific training to become “a pediatric research nurse” is included in the C4C Academy platform. Whatever the specialized training and at least in France, there is no recognition of their professional “research expertise” and no wage premium. Administrative and financial recognition of nursing excellence would help with conducting high quality research in high risk pediatric settings by increasing adherence to good clinical and laboratory practices (37, 38).
2) The time needed for evaluation of the PIP at EMA, may potentially delay trial finalization and validation. Next, approvals from regulatory competent authorities and ethics boards at the national, European or international levels are needed. Unfortunately, we were not able to analyze the time needed for trial evaluation by the ethical and regulatory authorities as submission of all documents depends on the sponsor. Then the time needed for administrative and financial agreement and trial procedures at the level of the center may again delay trial initiation. In our experience with multicenter—multinational trials, this key step is often closely dependent on the knowledge of administrative steps and management of financial issues, that may delay or even block trial initiation (29). However, a worrisome trend is seen with regards to the increased amount of time between the administrative and financial steps, the initiation of the trial and the first subject inclusion at our site between periods 1 and 2. Clearly, more time was needed for each of these steps for a trial initiation in period 2 as compared to period 1, particularly for non-industrial trials. Similarly, increased time was needed during period 2 between the last visit of the last participant included at our site and trial closure by the sponsor. Nevertheless, we do not note an increase in overall trial duration at the CIC-RDB when comparing the two periods.

Approvals by competent authorities and ethics boards is a key step, which depends on the sponsor and with limited impact, if any, of the researchers when it comes to delays for approval. In the coordinator’ experience, delay/blockage in trial initiation require multiple interventions by the research team to solve all complex administrative steps (particularly if multinational trials). Areas for improvement could include increased collaboration and reactivity of the supervision authorities, reduction in the number of multiple administrative layers and clarification of all financial supports and expenses.

The analysis of our activity covers a long period of 23 years and has some weaknesses. It reports on the pediatric clinical trials conducted at a single pediatric clinical investigation center. The patients recruited for inclusion in the clinical studies are principally composed of patients under the care of the hospital practitioners. Although Robert Debré University hospital is one of the largest pediatric hospitals in France with the presence of most medical, surgical and psychiatric sub-specialties, we cannot exclude a bias of selection by the pharmaceutical industry and/or preferential recruitment by the investigators. For example, our collaborations with neonatologists and the involvement of our team in neonatal pharmacology for many years including the management or participation in important EU projects allowed for a significant but probably “center-specific” increase in drug evaluation in neonates, through local and multicenter trials (30, 31, 39–44).

A second limitation is the difficulty in determining the best cut-off for the evaluation of the effects of the European Pediatric Regulation which entered into force in 2007. We chose to study two periods (1998–2006 and 2007–2020) of unequal length, for the following reasons: (1) a transition period before and after the entry into force of such an important regulation is expected, the length of which is difficult to determine, (2) the years immediately preceding the Pediatric Regulation are likely to have been influenced by regulatory changes in the United States, (3) the full effects of the application of the Pediatric Regulation inevitably were not immediately detectable. We do note a steady increase in the number of open trials since 2010, consistent with the increased number of pediatric clinical research observed by other studies. In addition, the mean number of visits per inclusion included was 3 fold higher in 2020 than in 1998, illustrating increased trial complexity and involvement of patients and their families.

## Conclusion

During 23 years, we conducted a significant number of pediatric clinical trials, increased the expertise and competences of our team, trained many young investigators and research technicians and organized the first French Clinical Investigation Network (45). During this long period, we have seen profound changes in pediatric trial characteristics and in regulatory requirements. We observed that following the Pediatric Regulation, the number of pediatric industrially sponsored trials increased. Nowadays, the pediatric and clinical research communities realize the urgent need to support networks of pediatric clinical research centers, to develop specific pediatric training of all professionals involved in pediatric research, and to ensure sustained financial support for all initiatives, including the recently obtained for the European IMI project C4C (Conect4Children) (23).

## Collaborative CIC1426 Investigator Group of Robert Debré University Hospital

Investigators are the main pediatricians who participated in trial design and/or selection, in recruitment, and in patients’ follow-up and care: Pr. Auvin, Pr. Delorme, Dr. Vantalon, Dr. LecenDr.eux (Neurology and psychiatry), Pr. Carel, Dr. Tubiana Dr. Bismuth (Endocrinology), Pr. Baruchel, Pr. Dalle (Hematology, Hemato-oncology), Pr. Deschenes, Pr. Hogan, Dr. C. Dossier, Dr. Kwon (Nephrology), Pr. Faye, Pr. Meinzer, Dr. Melki (General Paediatrics - Immunology-Infectious diseases), Pr. Houdouin, Dr. Munck, Dr. Gerardin (Pneumology), Pr. Hugot, Pr. Vialla (Gastro-enterology), Pr. Biran (Neonatology), Pr. Schmitz (Obstetrics), Pr. El Ghoneimi (Surgery), Pr. Dauger (Paediatric Intensive Care), Dr. Bourat (Dermatology), Pr. C. Alberti, Dr. S. Guilmin Crepon (URC), and Pr. O. Bourdon (Pharmacy).

## Data Availability Statement

The raw data supporting the conclusions of this article will be made available by the corresponding author, without undue reservation.

## Author Contributions

JA participated in data analysis and wrote the first version of the manuscript. FL developed the database, maintained in a prospective manner, and performed data analysis. FK are pediatricians running the Clinical Investigation Center, organizing trial setup, and collaborating with investigators for patients’ inclusion and follow-up. EJ-A initiated the work as director of the Clinical Investigation Center CIC1426, participated in data analysis, reviewed the different versions, and finalized the manuscript. All authors agreed on the final version of the manuscript.

## Conflict of Interest

The authors declare that the research was conducted in the absence of any commercial or financial relationships that could be construed as a potential conflict of interest.

## Publisher’s Note

All claims expressed in this article are solely those of the authors and do not necessarily represent those of their affiliated organizations, or those of the publisher, the editors and the reviewers. Any product that may be evaluated in this article, or claim that may be made by its manufacturer, is not guaranteed or endorsed by the publisher.
